# Robust Magnetoelectric Backscatter Communication System for Bioelectronic Implants

**DOI:** 10.21203/rs.3.rs-5463005/v1

**Published:** 2024-12-05

**Authors:** Fatima Alrashdan, Joshua E. Woods, Ellie C. Chen, Wendy Tan, Zhanghao Yu, Wei Wang, Mathews John, Lukas Jaworski, Drew Bernard, Allison Post, Angel Moctezuma-Ramirez, Abdelmotagaly Elgalad, Kaiyuan Yang, Mehdi Razavi, Jacob T. Robinson

**Affiliations:** 1 Department of Electrical and Computer Engineering, Rice University, 6100 Main St, Houston, TX, 77005.; 2 Texas Heart Institute, Houston, TX, USA; 3 Department of Bioengineering, Rice University, 6100 Main St, Houston, TX, 77005.; 4 Applied Physics Program, Rice University, 6100 Main St, Houston, TX, 77005.; 5 Department of Neuroscience, Baylor College of Medicine, 1 Baylor Plaza, Houston, TX, 77030.

## Abstract

Wireless communication technologies for bioelectronic implants enable remote monitoring for diagnosis and adaptive therapeutic intervention without the constraints of wired connections. However, wireless data uplink from millimeter-scale devices deep in the body struggles to achieve low power consumption while maintaining large misalignment tolerances. Here, we report a passive wireless backscatter communication system based on magnetoelectric transducers that consumes less than 0.3 pJ/bit and achieves less than 1E-6 bit error rate at a distance of 55 mm while tolerating a misalignment of 10 mm. Using this robust data uplink, we designed a wireless cardiac sensing node that can transmit electrocardiogram signals from the beating heart surface of a porcine model to a custom external transceiver using the magnetoelectric backscatter uplink. This reliable, near-zero-power communication method provides opportunities for next-generation bioelectronics to feature real-time physiological monitoring and closed-loop therapies while maintaining a small form factor and low power consumption.

## Introduction

Miniature wireless bioelectronics that can be implanted in a patient to continuously measure and transmit biomarkers in real-time enable improved therapeutic interventions. This is particularly important for medical conditions that require intermittent interventions that are difficult to predict, like epilepsy, transient ischemic attacks, or cardiac arrhythmias. However, wireless communication with small devices in the body is often a challenge. Wireless communication with these implants would ideally use miniaturized antennas, consume very little power, operate at depths of centimeters below the skin, and tolerate centimeter-scale misalignments with an external transceiver to support a reliable communication link. Depending on the clinical application, the exact requirements for size, power, depth, misalignment, and data rates can vary, but these features generally drive the user’s need for wireless communication with bioelectronic implants.

Active communication systems, particularly Bluetooth technology, are frequently used for applications requiring large transmission distances, but this comes at the expense of higher power consumption when compared to passive communication systems. Bluetooth chips are commercially available and often include a power amplifier and an ultra-high frequency (UHF) antenna to facilitate extended communication distances, usually > 100 mm. However, implants that integrate Bluetooth typically have large footprints ranging from 200–900 mm^2^ and are power-intensive, consuming more than 15 mW of power during data transmission^1^.

Backscatter communication techniques rely on reflecting energy back to the transceiver and thus consume very little power from the implant but are often limited to shallow depths of only a few mm or narrow alignment tolerances also on the scale of a few mm. In one example, Lee, J. et al. used a 1 GHz radio-frequency (RF) backscatter communication link to transmit neural activities from 48 cortical microchips to an external transceiver^2^. However, electromagnetic waves at these high frequencies are subject to significant losses when traveling through biological tissues, hence restricting the application of this technology to shallow implants with an operational depth of less than 5 mm^2,3^ and requiring an additional implanted relay coil. On the other hand, ultrasonic backscatter systems can operate at much greater depths compared to RF backscatter, reaching 100 mm for miniaturized implants with areas of 13.5 mm^2 4^. However, focused ultrasound links are sensitive to misalignments of just 1 mm between the implant and the external transceiver, making it difficult to establish continuous long-term communication links.

Magnetoelectric (ME) technology has recently been proposed as a wireless power transfer and data downlink solution for miniaturized implants with the advantages of large misalignment tolerance and high energy density. Prior work has shown that mm-sized implants can receive up to 10 mW power at depths of 30 mm and with high misalignment tolerances^5–9^.

Given that the ME transducers support wireless power and data downlinks at relatively large depths and with high misalignment tolerances, we hypothesized that magnetoelectrics could also provide a means for wireless data uplink with similar advantages. We previously reported a passive backscatter communication system using ME materials that consume less than 5 nW on the implanted device; however, the operational depth was limited to 15 mm due to the low signal-to-noise ratio of the ME backscattered field and the shallow modulation depth of frequency modulation^10^. One solution to improve the operational distance is to use signal processing techniques to demodulate noisy signals^11^, but this comes at the cost of the transceiver system complexity and is often specific to the operating environment in which the system is trained or developed.

**Fig.1|:**
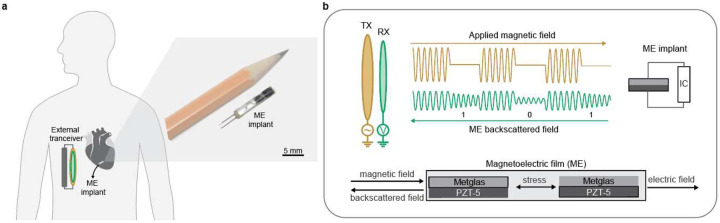
ME backscatter system overview. a) Wireless cardiac sensing using ME backscatter uplink. Right inset: a packaged ME cardiac sensor is shown next to a pencil to demonstrate the device size. b) Illustration of magnetoelectric backscatter communication uplink using a magnetoelectrics transducer that combines a magnetostrictive layer(Metglas) and a piezoelectric layer(PZT-5). The transducer is excited using a magnetic transmitter coil TX that generates a pulsed magnetic field. Upon excitation, the ME transducer generates a backscattered field that is detected using a magnetic receiver RX.

Here, we report a passive communication system that consumes less than 0.3 pJ/bit to support robust uplink communication from deeply implanted miniaturized ME bioimplants. Our system supports an operational range of 55 mm while maintaining a bit error rate (BER) of less than 1E-6. Compared to previous ME passive communication systems, this represents an improvement of 250% in terms of operating depth and more than 80 times in terms of BER^10,11^. Furthermore, our system exhibits a remarkable tolerance to translational misalignment where the low BER is maintained with a misalignment of more than 10 mm at a depth of 55 mm. To achieve these results, we 1)designed a sensitive receiver that can detect weak ME backscattered magnetic fields as small as 10 nT, 2)improved the modulation depth of the backscattered signal using optimal loading, and 3)implemented adaptive transceiver gain control. As a proof-of-concept, we demonstrate ME backscatter communication with a wireless cardiac sensor (Fig 1) that can detect and stream real-time intracardiac electrograms (EGMs) from a beating porcine heart despite centimeter-scale movements that are the result of cardiac contraction and respiration.

## Magnetoelectric backscatter communication

To create passive ME backscatter communication systems, we capitalize on two key features of the ME material. First, ME transducers generate a backscattered magnetic field in response to external magnetic excitations. We use this field as a carrier signal. Second, ME transducers’ effective mechanical properties can be tuned by an external electric load. We leverage this feature to modulate the amplitude of the carrier signal to encode data.

**Fig.2|:**
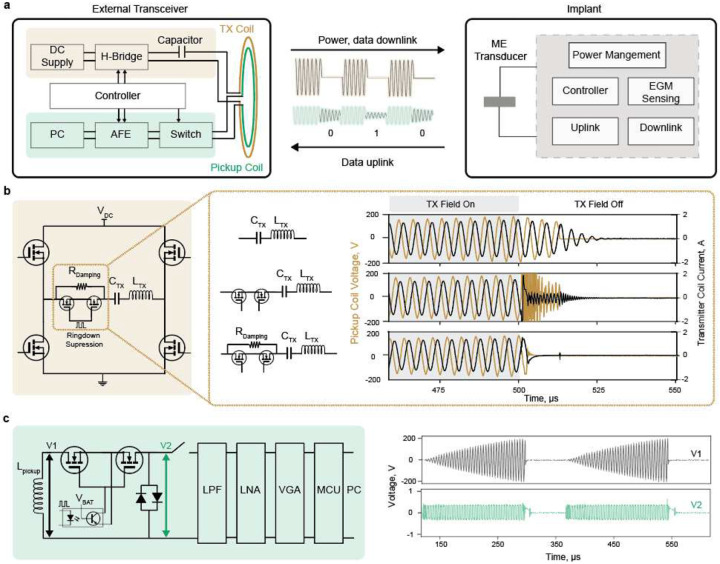
ME backscatter external transceiver design. a) Overview of the ME communication system components. The proposed system combines a custom external transceiver (Supplementary Fig1) and an mm-sized magnetoelectric implant. The power and downlink data are wirelessly delivered through the ME link; the ME backscatter is exploited for uplink communication. b) Transmitter ringdown suppression solution. The transmitter resonance circuit is powered by an H-bridge. To avoid cross-coupling between the transmitter excitation field and the backscattered field during the ringdown period, the transmitter ringdown is suppressed by adding an AC MOSFET in series to switch a 330-ohm damping resistor. c) ME backscattered field receiver circuit. The pickup coil is isolated from the detection circuit through a high-voltage AC switch during the excitation field. Once the field is off, the pickup coil is connected to the front-end detection circuit that combines a low pass filter(LPF), low noise amplifier (LNA), and variable gain amplifier (VGA) to amplify and filter the measured signal.

The ME transducer consumes no power to generate the backscattered field; rather, the power is consumed by the external transmitter that creates the excitation field. While this feature enables data uplink from miniaturized ME implants at near-zero power from the implant, the fact that the backscattered field is driven by the excitation field implies that the transmitted field and the backscattered field oscillate at the same frequency. This makes it difficult to extract the relatively weak backscattered field that contains data from the large transmitter field that powers the implant. To mitigate this limitation, we detect the “ringdown backscattered field,” which is the magnetic field that is created by the residual energy in the resonant ME material after the excitation field is turned off (Fig. 2a)^10^.

A key challenge in detecting the ringdown ME backscattered field is the difficulty of designing a transmit (TX) / receive (RX) system that can efficiently generate a magnetic excitation field and accurately measure backscattered signals. Resonant transmitter systems generate magnetic fields at significantly higher efficiency than non-resonant systems. However, a resonant transmitter coil (TX coil) stores energy during the excitation field that needs some time to be dissipated after the field is turned off. As a result, it can be difficult to detect the backscattered field right after the transmitter power is turned off because the residual energy in the TX coil creates a magnetic field that interferes with the ability of the RX coil to distinguish the ringdown backscattered signal from residual TX signal.

To overcome this challenge, we implemented a transmitter ringdown suppression circuit that rapidly dissipates the TX coil’s stored energy (Methods). Fig. 2b shows that by leveraging this design, the TX coil current is completely silenced a few microseconds after the field is turned off, and the ringdown backscattered field can be reliably detected.

**Fig.3|:**
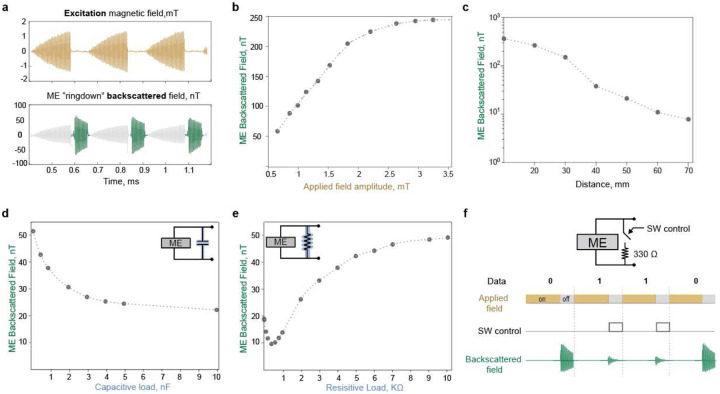
Backscattered field characteristics. a) Measured ME ringdown backscattered signal using the proposed external transceiver. The TX coil generates a pulsed magnetic field to excite the ME transducer. When the TX is turned off, the TX coil is silenced thanks to the ringdown suppression circuit. The RX coil detects the ME ringdown backscattered field. b) ME backscattered field as a function of the excitation magnetic field intensity. The amplitude of the excitation field was increased from 0.5 −3.5 mT while the frequency was fixed at 218kHz. The excitation field was superimposed on a 6.5mT DC field generated using a permanent magnet. c) ME backscattered field as a function of transducer-TRX distance. The ME transducer is placed at different distances from the center of the TRX coils. The excitation field at zero distance from the coil is set to 2 mT. In addition, a DC bias field of 6.5 mT was applied using a permanent magnet. d) ME backscattered field as a function of the capacitive load. Sweeping the capacitive load connected in parallel across the ME transducer changes the ME backscattered field. e) ME backscattered field as a function of the resistive load. Sweeping the resistive load connected in parallel across the ME transducer changes the ME backscattered field. f) ME load modulation induces backscattered field amplitude modulation. Switching the load across the ME transducer between an open circuit and 330-ohm load results in 0.6892 modulation depth of the backscattered field amplitude. Hence, to encode digital 0, the ME transducer is kept open-circuited; to encode digital 1, the transducer is connected to a 330-ohm resistive load.

Detecting the ringdown backscattered field signal requires a magnetic sensor that can achieve sufficient sensitivity to detect the weak ME backscattered field without saturating when exposed to the strong excitation field. These requirements rule out most commercially available sensors like Superconducting Quantum Interference Devices (SQUIDs), Giant magnetoresistance (GMR), and Hall effect sensors as they either lack the required sensitivity, bandwidth, or risk saturation under strong excitation field^12–14^. Differential pick-up coils are a potential solution, as they can cancel the effect of the excitation field if perfectly aligned with the TX coil. However, they are only sensitive when the ME transducer is positioned off-center from the TX coil, where the excitation field is weaker, limiting the operation distance and misalignment tolerance. Moreover, the off-center operation usually requires higher transmission power, reducing the system’s overall efficiency and raising concerns about the magnetic field safety limits.We designed a single pickup coil (RX coil) that is concentric with the TX coil to enable more sensitive detection and larger misalignment tolerance. The RX coil is connected to an analog front-end circuit (AFE) for signal processing and data demodulation, as depicted in Fig. 2c, and can detect fields as low as 10 nT (Methods). A significant challenge here, however, is that the strong inductive coupling between the TX and RX coils induces a large voltage across the RX coil that can reach 400 VPP with < 2mT at the TX coil. To isolate the pickup coil from the AFE circuit when the excitation field is on, we designed a high-voltage AC switch using back-to-back MOSFETs (Methods) as off-the-shelf switches either don’t have high voltage compliance (active switch ICs are limited to <100 Vpp) or are slow. The external transceiver synchronizes the operation of the transmitter and receiver to generate excitation fields and record ME ringdown backscattered fields as shown in Fig. 3a.

One of the main challenges in establishing a passive ME communication link is the decay of the received backscattered field as the ME transducer moves away from the external transceiver (TRX). There are two main factors driving this behavior: 1) the strength of the *excitation* field decays as the distance from the TRX increases; hence, the ME transducer experiences a lower excitation field that results in a weaker backscattered field (Fig. 3b), and 2) the strength of the *backscattered* field decays as the distance to the TRX increases; because the TRX is farther from the source of the backscattered signal. As shown in Fig. 3c, the ringdown backscattered field received by the RX coil decays from 250 nT at 20 mm to 10 nT at 60 mm. In addition to the excitation field strength, the backscattered field also depends on the excitation field frequency and the DC bias (Supplementary Fig2,3).

To receive a reliable ME backscatter signal during the implant-TRX distance and alignment change, we designed an adaptive variable gain control (VGA) algorithm that dynamically adjusts the gain in response to the strength of the received magnetic field (Methods). The movement of the transducer will likely result in data transmission disruption; for example, assuming the cardiac device will operate at a distance of > 30 mm from the TRX, we set the VGA gain to 35 dB to ensure a high signal-to-noise ratio. However, as the heart beats and the device gets closer to the TRX, the ME backscattered signal will likely saturate the VGA output. If, on the other hand, we set the VGA gain to 10 dB to avoid saturation at a close distance, the received signal will be very weak as the device moves away. Hence in both cases, the data cannot be demodulated correctly, as digital 0 and digital 1 can no longer be distinguished. With the adaptive gain, however, the external TRX can automatically adjust the VGA gain to overcome this limitation.

To encode digital data using the ME transducer, we found that an optimal resistive load connected to the ME material produced the strongest modulation of the ring-down amplitude. Changing either the capacitive load or the resistive load across the ME transducer changes the amplitude of the backscattered field. With capacitive load modulation, the maximum modulation depth is 0.3565, achieved when switching between an open circuit and short circuit conditions (Fig.3.d). In contrast, resistive load modulation at an optimal resistive load of 330 ohms (Fig.3.e) produced a modulation depth of 0.6892. This is 75 times higher modulation depth compared to the previously reported frequency modulation scheme in^10^. Hence, to encode digital 0 and digital 1, we switch the load across the ME transducer between an open circuit and 330 ohm, respectively (Fig.3.f). The energy consumed to transmit one bit is effectively the energy required to switch the load across the transducer, which is calculated as 0.235 J (Methods).

## System performance

When we evaluated this communication scheme over different operational distances, we observed a Bit Error Rate (BER) of less than 1E-6 over a wide lateral misalignment range of 10 mm at a depth of 55 mm. BER is a standard metric to evaluate the reliability of communication systems, with lower values reflecting higher reliability. This is directly dependent on the strength of the carrier signal and the modulation depth; if the received signal is strong enough and there is a significant difference in the amplitude at the different loading conditions, then the digital 0 and 1 can be directly distinguished.

**Fig.4|:**
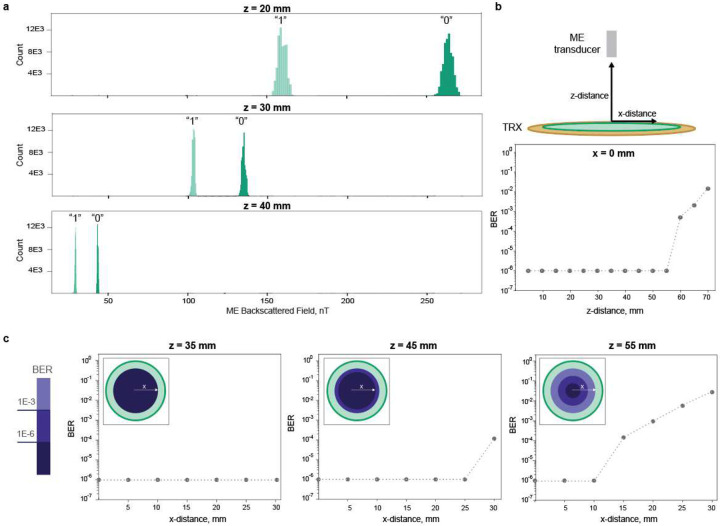
ME backscatter system performance. a) Distribution of ME backscattered field amplitude for digital ”0” and digital ”1”. Tests were conducted by sending 131,000 at different TRX-implant distances of 20 mm, 30 mm, and 40 mm. b) Measured BER versus the external TRX-implant distances. The BER was measured by sending 130,000 PRBS samples at different TRX-implant distances. c) Measured BER as a function of translational misalignment. The BER is measured at 0 to 30 mm x-distance misalignment from the coil center at TRX-implant distances of 35 mm, 45, and 55 mm.

The ME backscatter communication system can transmit data over a distance of 55 mm with a low BER of less than 1E-6. The histograms in Fig. 4a. clearly show the difference between the backscatter field amplitude representing the “digital 0” and “digital 1”. As the transducer moves away from the TRX, the backscattered field gets weaker, and the separation between “digital 1” and “digital 0” gets smaller. However, we can still distinguish between the two signals, hence correctly demodulating the received data at deep distances. The BER value is measured for different transducer-TRX distances, as seen in Fig. 4b. A BER of < 1E-6 is maintained for an operation distance of 55 mm, sufficient for many bioelectronic applications.

The ME backscatter uplink enables data rates of up to 6.5 kBps with BER < 1E-6. To communicate one bit using the proposed ME link, the excitation field should be turned on enough time to activate the film and then turned off for a long enough time to record the backscattered field. The tradeoff here is that a longer on-time means stronger backscattered field value since more energy is stored in the ME transducer; however, it also means lower data rate values. Furthermore, since we use a resonance transmitter coil, there is a ”ring-up” time until the transmitter reaches the maximum excitation value. We evaluated the BER at different data rates, where we set the field off-time to 50μs and swept the on-time from 300 to 50 μs (Supplementary Fig. 4).

The ME backscatter uplink has a 10 mm translational misalignment tolerance, even at a depth of 55 mm. To test the robustness of our system against expected misalignment, we placed the ME transducer at different z-distances. We moved it gradually from the center toward the edge of the pickup coil. Since we use a circular coil, this will be symmetric across different directions. As seen in Fig. 4c, the system maintains low BER even when the implant is more than 10 mm shifted from the center. This is higher than the misalignment tolerance reported for ultrasound communication, where a near-perfect alignment (< 2 mm shift) is required to transmit data.

## In vivo demonstration of wireless cardiac sensing

The expanded depth and misalignment tolerance of the passive ME uplink allowed us to receive real-time intracardiac signal recordings from millimeter-sized ME sensors placed on the surface of a beating pig heart. These sensors integrate an 8 mm × 2.5 mm ME film with a custom printed circuit board (PCB) incorporating power management circuitry, electrogram (EGM) sensing (AD8233), and bidirectional communication modules, as shown in Fig. 2a. The packaged sensor (Fig. 1a) is 2.5 cm long and 3 mm in diameter; these dimensions are chosen to enable delivery with a 12 Fr (~4 mm diameter) catheter or a smaller catheter for future studies.

**Fig.5|:**
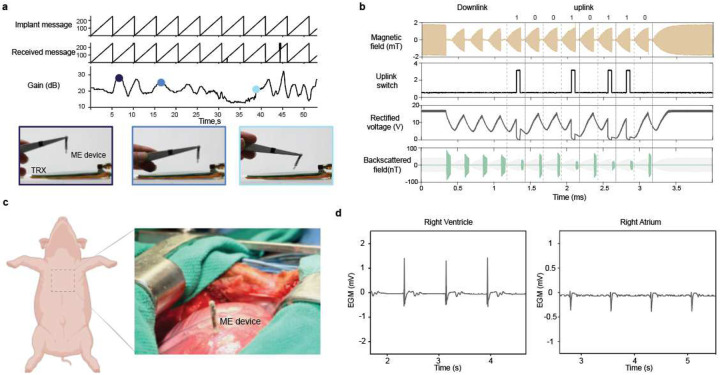
In vivo demonstration of ME backscatter communication. **a)** Demonstration of adaptive gain control. Different instances from supplementary video 1 capture the TRX capabilities to receive the implant’s message under different distances and misalignment scenarios. The VGA gain changes dynamically according to the implant location. **b)** Communication protocol for the cardiac ME sensor. Once the device receives a downlink message to initiate EGM recording followed by an 8-bit uplink message 10010110**. c).** The surgical site of ME cardiac implant placed on the heart of a porcine model using needle recording electrodes. The metal retractor is replaced with a PVC retractor to reduce electromagnetic interference(supplementary fig 5). **d)** EGM signals are recorded using an ME sensor placed on the right ventricle and right atrium of a porcine model’s beating heart and transmitted wirelessly through the ME uplink

One of the main challenges for wireless, battery-free cardiac implants is tolerating continuous heart movement. To demonstrate the device’s capability of transmitting real-time data correctly despite continuous movement, we moved the device arbitrarily at different distances and orientations and showed that the TRX can still successfully receive the implant’s message. For demonstration, we programmed the implant to continuously transmit a sawtooth signal, as shown in supplementary video 1. Different instances of this demonstration are shown in Fig 5a. The gain is automatically adjusted based on the device location to ensure reliable data transmission.

In addition to data uplink, the device uses the same ME transducer to receive power and data from the external transceiver, thereby keeping the design compact. To control the device operation, the external transceiver sends a 3-bit downlink message encoded in the transmitter field’s timing, as detailed in our previous work^7^. The implant then encodes an 8-bit uplink message immediately after the downlink command to encode real-time data by changing the load across the ME transducer. For each bit, the magnetic field is turned on for 200 μs to excite resonance in the ME film and then turned off for 50 μs to measure the modulated backscatter data, as shown in Fig. 5b. To measure continuous biological data, a ‘sense’ command is sent at the desired sampling rate (100–200 Hz). For each ‘sense’ command, the device samples the output of the onboard bio-amplifier and encodes that data in the following uplink bits.

ME cardiac sensors placed on the epicardial surface of a beating pig’s heart can wirelessly transmit the EGM signal to the external transceiver. For this demonstration, we inserted the sensing node with two attached stainless steel needle electrodes into the right ventricle after exposing the heart through median sternotomy, as shown in Fig. 5c. Although the device has been designed for minimally invasive endovascular delivery, open heart surgery was used here to validate its robust operation and evaluate the quality of the recorded signal. The external transceiver was then positioned at the skin’s surface to power the implant and receive the recorded EGM signal wirelessly. Adopting the communication protocol presented in Fig. 5b, the implant receives a downlink message to initiate the recording and data transmission. As shown in Fig. 5d, we can record the EGM signal while the device is placed at the heart surface, experiencing dynamic movement associated with the heartbeats. The ventricular EGM shows two distinct peaks indicating ventricular polarization and repolarization phases. We then moved the device to the right atrium to capture atrial EGM signals. The EGM recording shows atrial depolarization and repolarization phases with smaller amplitudes than ventricular signals due to thinner atrial walls and different conduction characteristics of atrial tissue. These recordings provide localized measurements from specific areas of the heart. Hence, they provide precise information about the timing and sequence of the heart’s electrical activation, which is vital to diagnosing arrhythmias or other conduction disorders and can guide therapies such as cardiac resynchronization therapy.

## Conclusions

We have presented a robust, ultra-low-power communication solution for implantable medical devices utilizing magnetoelectric technology. The proposed system supports reliable data transmission from small implants to external transceivers over distances greater than 50 mm and a misalignment tolerance over 10 mm, which is sufficient for numerous medical applications. A key strength of this technology is its high reliability in the presence of misalignment, which makes it suitable for dynamic implants like cardiac and gastrointestinal applications. These capabilities are validated through in vivo demonstration of cardiac sensing of electrograms recorded from the porcine model’s heart surface that paves the way for continuous real-time monitoring for cardiac arrhythmia diagnosis. While this work primarily focuses on bioelectronics, the developed system can be utilized in other domains where power and size constraints are critical, including the Internet of Things and identification systems. However, applications that require high data rates may be better served with other solutions like ultrasound or radio frequency. Additional work to create multi-implant networks and enable full-duplex communication would improve the data transmission efficiency and the system’s data rate to support next-generation bioelectronics that feature adaptive and connected systems for more effective and personalized therapies.

## Methods

### Magnetic transmitter design

The magnetic field transmitter generates a pulsed AC magnetic field to activate the ME transducer using a resonance TX coil powered by an H-bridge based on the APEX Microtechnology SA310 component. The frequency of the generated field is adjusted to match the ME transducer’s resonance frequency of 218 kHz by tuning a series capacitor connected to the TX coil. The TX coil is a two-layer circular spiral coil with an inner diameter of 4 cm and an outer diameter of 9.5 cm. The coil uses 18 AWG litz wire (MWS Wire) to minimize the skin effect. The transmitter ringdown circuit is implemented by connecting an AC switch of two back-to-back MOSFETs in series with the resonant coil to shift the frequency during the field-off time. During the on-time of the magnetic field, the switch is closed, and the excitation current usually flows through the resonance coil. We used IMW120R350M1H CoolSiC MOSFETs with a low on-resistance of < 50 mohm to maintain the system efficiency. When the field is turned off, we immediately open the switch; due to the switch’s parasitic capacitance, the coil’s resonance frequency is shifted to > 1 MHz, resolving the interference issue. To suppress these oscillations and avoid potential amplifier saturation problems, we added a parallel damping resistor of 330 ohms. This resulted in critical damping conditions of the overall RLC circuit, absorbing the stored energy much faster.

### Backscattered field receiver design

We designed a large circular spiral printed circuit board (PCB) pick-up coil with an 8 cm outer diameter and 68 turns. To create a high-voltage AC switch, we used two series of N-channel MOSFETs (IMW120R350M1H CoolSiC). When the excitation field is on, the MOSFETs are turned off to isolate the pickup coil and the AFE. When the magnetic field is turned off, the MOSFETs are switched to connect the pickup coil and the AFE. We used an optocoupler with a fast switching speed to drive the MOSFETs gates to ensure proper isolation of the ground of the control and power circuit. Moreover, we used a pair of diodes to limit the voltage, which ensures safe voltage levels for the electronics and a negligible reflected load on the TX coil. The AFE circuit has an enable switch, a fourth-order active low pass filter (LTC1563), a low noise amplifier (LNA) (AD8432), and a variable gain amplifier (VGA) (AD8338). The gain of the VGA ranges from 0 to 80 dB and is manually adjusted using a digital-to-analog converter (DAC) based on the depth and location during the experiments in Fig3–4 to avoid saturation and ensure correct data demodulation. The switch timing of the entire system was controlled with a high-speed microcontroller (MCU) (NXP LPC54005). The microcontroller’s 12-bit analog-to-digital converter (ADC) samples the VGA output during ringdown to demodulate the received message. We implemented a peak detection algorithm on the MCU that detects the peaks, averages the value, performs threshold detection, and transmits the data through the serial UART communication to a computer. The receiver limit of detection is calculated based on the noise level of the VGA output in the absence of an ME transducer. The field corresponding to this voltage (VGA gain = 15 dB) is around 1.4 nT, so the system should detect fields > 10 nT for a conservative estimate.

### Magnetoelectric transducer fabrication

To fabricate the ME transducer, we prepared a 30*30 mm ME sheet by sandwiching a 267um-thick PZT-5H layer (Piezo Systems) between two 25um-thick Metglas 2605SA1 layers(Metglas Inc) using M-bond epoxy(M-Bond 43-B). We then used a femtosecond laser to cut the sheet to the required transducer size. We used 7.5*3 mm films for benchtop testing. For the cardiac sensing device, we used 8*2.5 mm films. In general, the resonance frequency of ME transducers -the carrier frequency- –can be calculated as $f_{r}\sim \frac{1}{2L}\sqrt{\frac{E_{eq}}{\rho_{eq}}}$ where L: length, E: effective young modulus, and ρ: effective density. Hence, for mm-sized Metglas-PZT-5 transducers, this value ranges from 100–500 kHz, where the tissue absorption is minimal.

### Backscattered field measurement setup

To test the characteristics of the ME backscattered signal, we used a 7.5*3 mm ME film that has a resonance frequency of around 218 kHz. The film is excited by the TX coil that generates a controlled magnetic field at 218kHz. To detect the backscattered field signal, we recorded the output of the VGA using a PCIe-6374 National Instruments (NI) data acquisition (DAQ) system, which was interfaced with customized MATLAB code. The field is calculated using the following equation: $B_{ME}=\frac{VGA\;\text{output}}{\left(VGA\;\text{gain}*\;LNA\;\text{gain})*(2*\pi *f*A*N\right)}$ where A is the pickup coil average area, and N is the number of turns. During each trial, we turn the magnetic field for 250 μs to excite the ME film, then we turn it off for 50 μs to record the the backscattered field. We implemented a peak detection algorithm in MATLAB to identify the peaks of the backscattered signal during the ringdown period of each trial. We then average these peaks over five consecutive trials to ensure accuracy. For energy per bit calculations, the energy required to encode one bit is the energy consumed to switch the load across the transducer. Since we use a MOSFET to switch the load, this can be calculated as the energy consumed to switch the MOSFET gate /bits = 0.5*Cgate*Vgatê2 /bits= 0.5*280*3.3^2 /6500 = 0.235 pJ/bit, assuming 6.5kbps data rate.

### BER measurement setup

To measure the BER, we used PCIe-6374 NI DAQ interfaced with a customized MATLAB code to generate 130,000 samples of pseudo-random bit sequence (PRBS). The PRBS signal is used to switch the load across a 7*3.5 mm ME transducer between an open circuit (bit 0) and 330 ohm (bit 1). The excitation field is tuned to 218 kHz to match the transducer’s resonance frequency while its amplitude is set to 2.6 mT( measured at the TX coil surface). This value is far below the safety limit of 8mT for the 218 kHz. The backscattered signal is received using the system described above. The data is demodulated using an averaging threshold.

### Adaptive VGA gain control

The gain of the VGA is adjusted based on the implant depth and location. To do that, the MCU records the LNA output during the first downlink bit. Since the LNA has a fixed gain of 16 dB, it is unlikely to saturate even when the device is at zero distance from the TRX. The LNA output represents the level of the backscattered field value; hence, we can use it to set the gain of the VGA. This value is used to calculate the gain that will result in maximum VGA output without saturation. The MCU sets the new gain during the third downlink bit before the data uplink starts.

### Surgical preparation and procedure

Animals are sedated via an IM injection of Telazol 2–6 mg/kg and Atropine Sulfate 0.02–0.05 mg/kg. Then, Isoflurane is administered via facemask to induce general anesthesia for surgical interventions. Pigs are intubated and appropriately prepared for surgery. IV catheters are established, and the animals are transported to the OR. Naxcel (Ceftiofur) 3–5 mg/kg (or equivalent) is administered IM. Appropriate preoperative analgesics such as Buprenorphine HCL 0.005–0.1 mg/kg and/or Flunixin Meglumine 1.1–2.2 mg/kg IM, IV or SQ. (or equivalent to either) are administered. For this specific study, the anesthetic of choice is isoflurane (0.5–5.0%) and/or propofol (10–20 mg/kg/hr IV).

The animal is placed on the table in the dorsal position. The chest is aseptically prepped with betadine. A midline incision is performed from the sternal notch to the xiphoid process using electrocautery. Once all of the soft tissues have been divided, the sternum is opened using a sternal saw, followed by an oscillating saw for the manubrium. The sternum is then retracted using a self-retaining retractor made out of PVC. With the heart on sight, the thymus is resected, and the pericardium is opened with a T incision. Silks are used to create the pericardial cradle and stabilize the heart.

## Supplementary Material

1

## Data Availability

The main data supporting the results of this study are available in the paper and its Supplementary Information section.
