# Supplementary material for: Robust Magnetoelectric Backscatter Communication System for Bioelectronic Implants
Source: Res Sq. 2024 Dec 5:rs.3.rs-5463005. Preprint. [Version 1] doi: 10.21203/rs.3.rs-5463005/v1 (PMC11643333; doi:10.21203/rs.3.rs-5463005/v1)
Supplement: 1 [file NIHPPrs5463005V1-supplement-1.pdf]

Supplementary figures

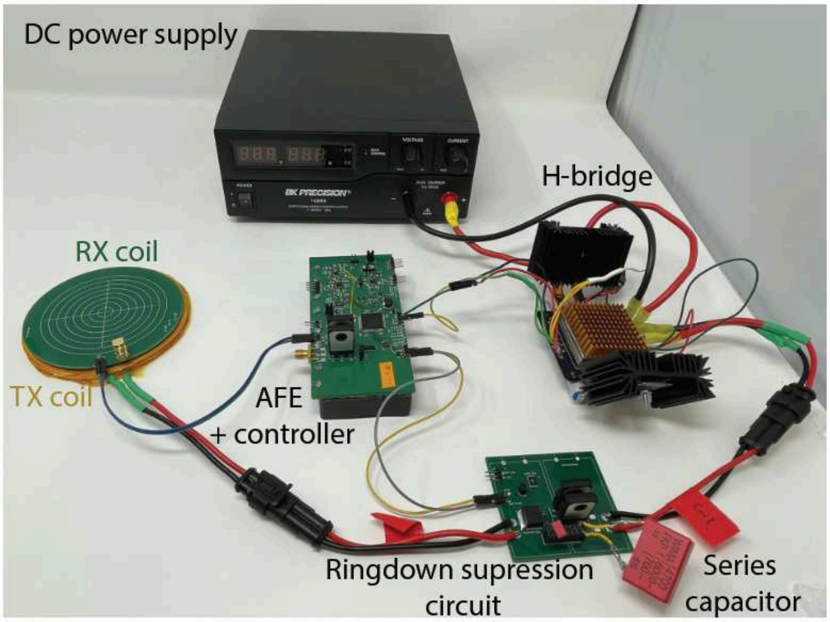

Supplementary Fig1: Prototype of the custom external transceiver. A DC power supply powers an H-bridge to drive AC current into the resonant transmitter that combines a series of capacitors and a TX coil. A ringdown suppression circuit is implemented to eliminate the interference between the ME backscattered signal and the TX ringdown field. The ME backscattered signal is measured using an RX coil that is connected to an AFE board to demodulate the uplink data. The AFE board can be connected to a computer to read the data.

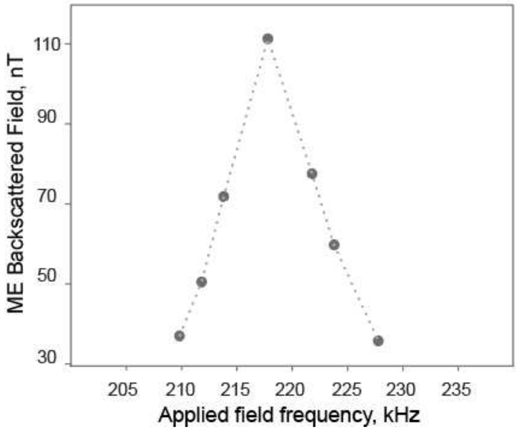

Supplementary Fig2: ME backscattered field as a function of the excitation magnetic field frequency. The backscattered magnetic field is measured at different excitation field frequencies during the ringdown period. The excitation field amplitude was maintained at 1.1 mT while the ME transducer was placed at a distance of 25 mm from the pickup coil. In addition, a DC bias field of 6.5 mT was applied using a permanent magnet.

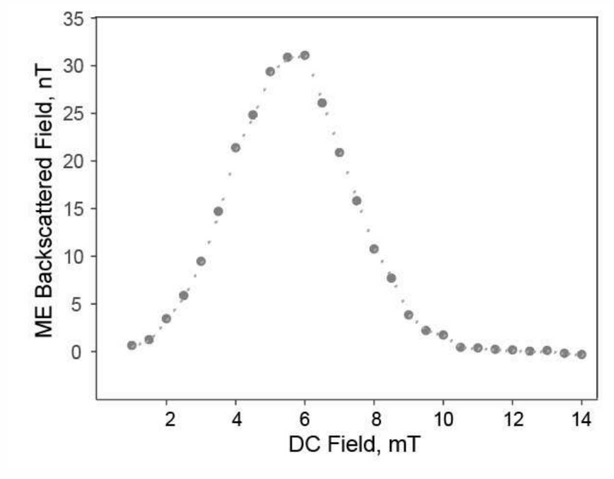

Supplementary Fig3: ME backscattered field as a function of the DC bias field. The ME transducer is subjected to a fixed AC excitation field at 218 kHz and a variable DC bias voltage that is applied using a permanent magnet. The intensity of the DC bias was controlled by changing the distance between the ME transducer and the DC magnet. A DC magnetic sensor is used to measure the DC bias at the transducer location.

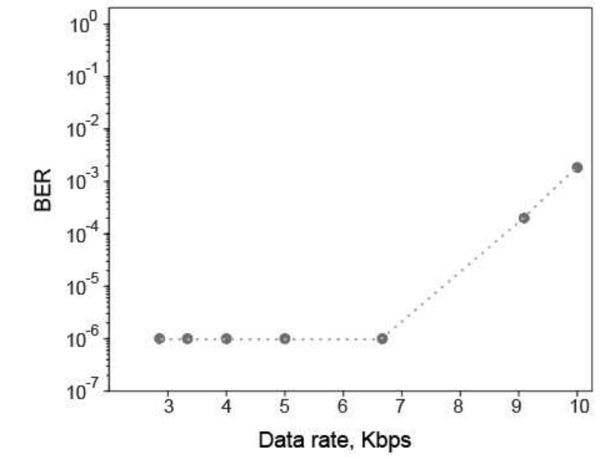

Supplementary Fig4: Measured BER as a function of the data rate. The BER is measured by transmitting 130,000 PRBS samples from the implant while placed at 35mm from the TRX. toff is set to 50µs while ton is swept from 300 to 50 µs with 50 µs step.

Supplementary video

Supplementary video 1: Demonstration of ME backscatter uplink communication reliable performance across different depths and misalignments.
